# APP/Aβ Signaling Orchestrates Reactive Astrocyte Networks in Alzheimer's Disease

**DOI:** 10.1111/jnc.70526

**Published:** 2026-07-24

**Authors:** Gretsen Velezmoro Jauregui, Sophie Coomes, Elizabeth Emmett, Alex Thatcher, Nicole Oyelakin, Maria Čarna, Jana Zelinkova, Jan Sebastian Novotny, Natalie Polakova, Tarun Kuruvilla, Robert Zorec, Alexei Verkhratsky, Vladimir Parpura, David Morgan, Kenneth L. Moya, Clara Limback‐Stokin, Gorazd Bernard Stokin

**Affiliations:** ^1^ International Translational Neuroscience Research Institute Zhejiang Chinese Medical University Hangzhou Zhejiang China; ^2^ School of Medicine University of Bristol Bristol UK; ^3^ Fritchie Centre, Gloucestershire Health and Care NHS Foundation Trust Cheltenham UK; ^4^ Faculty of Engineering, Department of Bioengineering Imperial College London London UK; ^5^ Central European Institute of Technology, Masaryk University Brno Czech Republic; ^6^ Institute for Molecular and Translational Medicine, Faculty of Medicine and Dentistry Palacky University Olomouc Olomouc Czech Republic; ^7^ Laboratory of Neuroendocrinology ‐ Molecular Cell Physiology, Institute of Pathophysiology, Faculty of Medicine University of Ljubljana Ljubljana Slovenia; ^8^ Celica Biomedical Ljubljana Slovenia; ^9^ Faculty of Biology, Medicine and Health The University of Manchester Manchester UK; ^10^ Department of Forensic Analytical Toxicology, School of Forensic Medicine Chinese Medical University Shenyang China; ^11^ Department of Geriatrics, Seventh Affiliated Hospital of Sun Yat‐Sen University Shenzhen China; ^12^ Department of Translational Neuroscience, College of Human Medicine Michigan State University Grand Rapids Michigan USA; ^13^ Center for Interdisciplinary Research in Biology, College de France, CNRS, INSERM Université PSL Paris France; ^14^ Neuropathology and Ocular Pathology Department Oxford University Hospitals NHS Foundation Trust Oxford UK; ^15^ Department of Neurology, Royal Gloucester Hospital Gloucestershire Hospitals NHS Foundation Trust Gloucester UK

**Keywords:** Alzheimer's disease, amyloid precursor protein, amyloid‐β peptides, reactive astrocytes

## Abstract

Alzheimer's disease (AD) is characterized by amyloid‐β (Aβ) accumulation, neurofibrillary pathology, synaptic dysfunction, and chronic neuroinflammation, yet the mechanisms driving early, localized pathology remain elusive. While traditionally viewed through a neuron‐centric lens, astrocytes express abundant amyloid precursor protein (APP)—predominantly Kunitz‐type protease inhibitor (KPI)‐containing isoforms—and possess the complete enzymatic machinery for APP processing and Aβ clearance. Astrocytic APP is a stress‐responsive signaling molecule regulated by inflammatory, metabolic, excitotoxic, and mechanical insults. Under local tissue stress, reactive astrocytes upregulate APP and shift toward amyloidogenic processing. The resulting bioactive fragments, including Aβ, promote astrocyte activation, disrupt homeostatic functions, and trigger feed‐forward upregulation of endogenous APP. We propose that this reciprocal coupling establishes a self‐reinforcing network where APP integrates local stress and diffusible Aβ propagates reactive states across the astroglial syncytium. This framework positions astrocytic APP signaling as an upstream driver of localized amyloid accumulation, neuroinflammation, and sporadic AD progression.

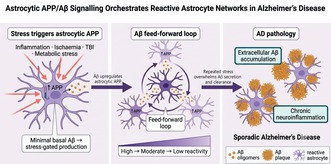

## Graphical abstract

Stress increases astrocyte APP production, generating amyloid‐β (Aβ). Aβ then diffuses to neighboring astrocytes, stimulating further APP and Aβ production and creating a self‐reinforcing cycle. Over time, impaired Aβ clearance leads to its accumulation, chronic inflammation, and contributes to sporadic Alzheimer's disease.

**Abbreviations**
AD: Alzheimer's disease
ADAM‐10: a disintegrin and metalloproteinase domain‐containing protein 10
AICD: APP intracellular domain
APH‐1: anterior pharynx‐defective 1
APLP1: amyloid precursor‐like protein 1
APLP2: amyloid precursor‐like protein 2
ApoE: apolipoprotein E
APP: amyloid precursor protein
AQP4: aquaporin‐4
Aβ: amyloid‐β peptides
BACE1: β‐site APP cleaving enzyme 1
BACE2: β‐site APP cleaving enzyme 2
cAMP: cyclic adenosine monophosphate
CNS: central nervous system
COX2: cyclooxygenase‐2
CSF: cerebrospinal fluid
EAAT1: excitatory amino acid transporter 1
ERK: extracellular signal‐regulated kinase
FDG: fluorodeoxyglucose
FOXO3: forkhead box O3
GFAP: glial fibrillary acidic protein
HIF‐1: hypoxia‐inducible factor 1
IDE: insulin‐degrading enzyme
IFNγ: interferon gamma
IGF: insulin‐like growth factor
IL‐10: interleukin‐10
IL‐12: interleukin‐12
IL‐18: interleukin‐18
IL‐1α/β: interleukin‐1 alpha/beta
IL‐6: interleukin‐6
IL‐8: interleukin‐8
iNOS: inducible nitric oxide synthase
JAK: janus kinase
JNK: c‐Jun N‐terminal kinase
KPI: Kunitz‐type protease inhibitor domain
LDLR: low‐density lipoprotein receptor
LPS: lipopolysaccharide
LRP1: lipoprotein receptor‐related protein 1
MCP‐1: monocyte chemoattractant protein‐1
MMP: matrix metalloproteinase
NADPH: nicotinamide adenine dinucleotide phosphate
NF‐κB: nuclear factor kappa‐light‐chain‐enhancer of activated B cells
NLRP3: NOD‐like receptor protein 3
NO: nitric oxide
OX2: MRC OX2 antigen
PEN‐2: presenilin enhancer 2
PET: positron emission tomography
PGE2: prostaglandin E2
PKA: protein kinase A
PKC: protein kinase C
PSEN1: presenilin 1
RNA: ribonucleic acid
ROS: reactive oxygen species
sAPPα: secreted α‐cleaved APP fragment
sAPPβ: secreted β‐cleaved APP fragment
STAT3: signal transducer and activator of transcription 3
TBI: traumatic brain injury
TDP‐43: TAR DNA‐binding protein 43
TFEB: transcription factor EB
TGFβ: transforming growth factor beta
TLR: toll‐like receptor
TNFα/β: tumor necrosis factor alpha/beta
TREM2: triggering receptor expressed on myeloid cells 2
YKL‐40: chitinase‐3‐like protein 1

## 1 Introduction

Alzheimer's disease (AD) is defined pathognomotically by extracellular amyloid‐β (Aβ) accumulation, intracellular neurofibrillary pathology, synaptic loss, and chronic neuroinflammation (Forloni and Balducci 2018; LaFerla and Oddo 2005; Pozueta et al. 2013). However, the mechanisms governing the early focal emergence and stereotypical progression of this pathology remain unresolved. Traditional neuron‐centric models position neuronal Aβ production as the primary driver of amyloid deposition (Masters and Selkoe 2012; Morris et al. 2014). Yet, these frameworks fail to fully account for the prolonged, clinically silent preclinical phase or the distinct regional heterogeneity characteristic of sporadic AD (Ferreira et al. 2020).

In response to diverse metabolic, inflammatory, and mechanical pathological stressors, astrocytes undergo complex transcriptional, morphological, and functional reprogramming represented by either reactive astrogliosis (Escartin et al. 2021) or astrocytic atrophy and asthenia. Notably, human neuroimaging and biomarker studies indicate that astrocyte reactivity is an early event that precedes overt neurodegeneration and gross amyloid deposition (Bellaver et al. 2023; Rodriguez‐Vieitez et al. 2016). This timing suggests that astrocytes are active drivers of early pathogenesis rather than passive bystanders responding to neuronal death.

Amyloid precursor protein (APP) provides a direct biochemical bridge linking early astroglial responses to classic amyloid pathology. Far from being restricted to the neuronal lineage, astrocytes robustly express APP—predominantly Kunitz‐type protease inhibitor (KPI)‐containing splice variants—and harbor the complete enzymatic machinery required for both Aβ generation and clearance (Haass et al. 1991; Jin et al. 2024; LeBlanc et al. 1991; Nakamura et al. 1992; Perez et al. 2010). Astrocytic APP expression is significantly elevated by physiological and pathological stressors; conversely, APP, its proteolytic fragments, and extracellular Aβ provoke astrocyte reactivity, establishing a self‐reinforcing signaling network (Apelt and Schliebs 2001; Kalaria et al. 1993; Rogers et al. 1999; Sugaya et al. 1998; Velezmoro Jauregui et al. 2024).

Whether astrocytic APP signaling functions as a primary driver of disease progression remains unknown. Here, we propose a testable model in which astrocytic APP acts as a stress‐responsive signaling hub linking local tissue injury to chronic amyloid pathology. In this framework, stress‐induced APP expression and processing promote sustained reactive phenotypes, while diffusible Aβ propagates APP‐dependent signaling to neighboring astrocytes. This model positions astrocytic APP signaling as an early driver of sporadic AD pathogenesis, offering a mechanistic hypothesis that integrates existing observations and generates experimentally testable predictions.

## 2 APP and Its Fragments in Astrocytes

**FIGURE 1:**
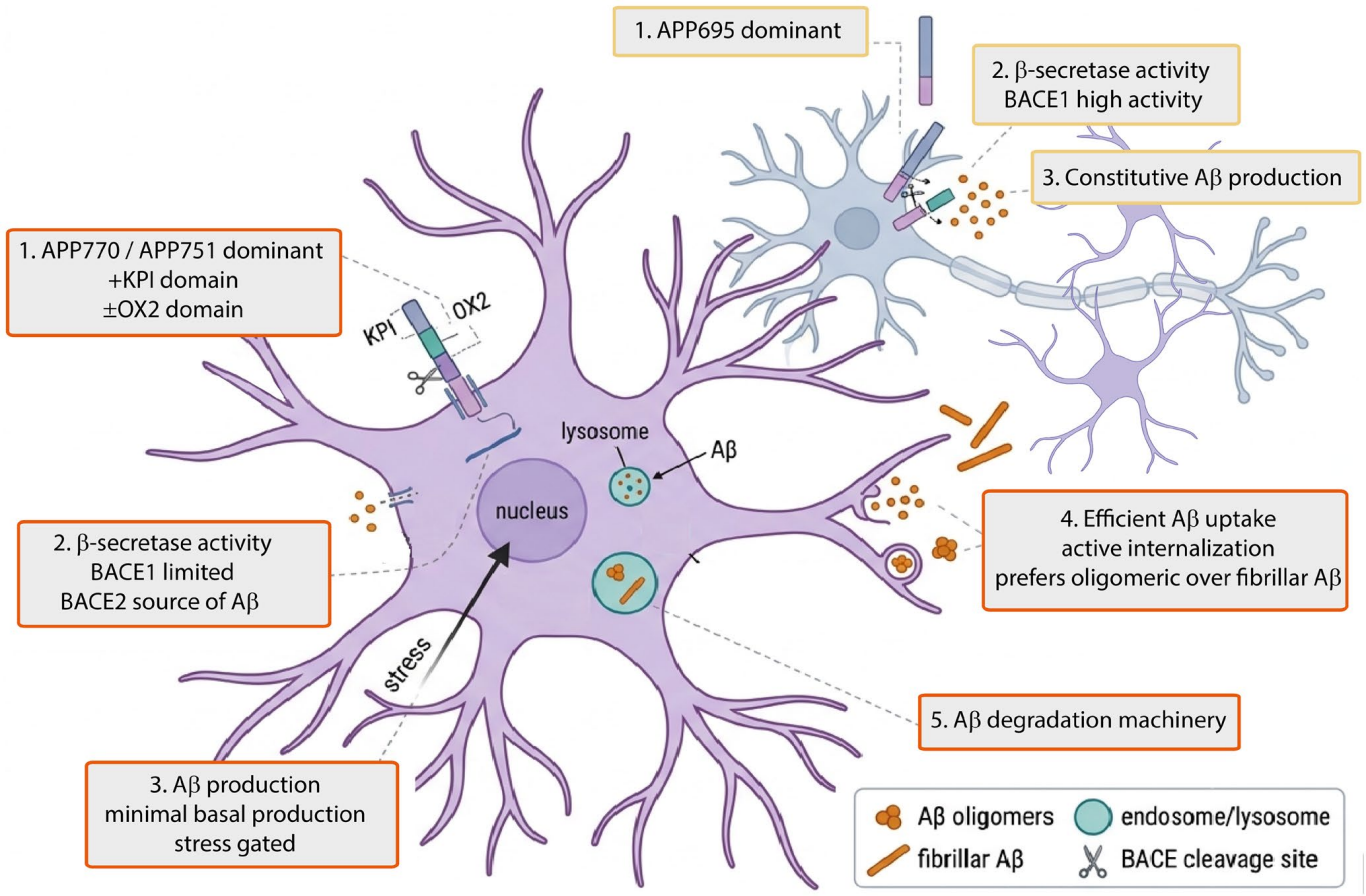
APP processing, uptake and degradation in astrocytes and neurons. Astrocytes predominantly express the KPI‐containing APP isoforms APP770 and APP751, exhibit low BACE1 expression, and probably little Aβ under basal conditions, although Aβ generation may increase during inflammatory stress. They also actively internalize and degrade extracellular Aβ. In contrast, neurons predominantly express APP695, exhibit high BACE1 activity, and constitutively generate and secrete Aβ. Aβ = Amyloid‐β peptides, APP = Amyloid precursor protein, BACE1 = β‐site APP cleaving enzyme 1, BACE2 = β‐site APP cleaving enzyme 2, KPI = Kunitz‐type protease inhibitor domain, OX2 = MRC OX2 antigen.

The APP is a type I integral membrane protein encoded by the *APP* gene on chromosome 21 (Goldgaber et al. 1987; Kang et al. 1987; St George‐Hyslop et al. 1987; Tanzi et al. 1987). Alternative splicing of exons 7 and 8 gives rise to three major variants: APP_695_, APP_751_, and APP_770_. These isoforms differ strictly by the inclusion or exclusion of the Kunitz‐type protease inhibitor (KPI) and the MRC OX2 antigen domains (Figure 1) (Tanaka et al. 1988).

### 2.1 APP Expression in Astrocytes

APP mRNA is robustly and constitutively expressed in astrocytes across species, including mice (Ohyagi et al. 1990), rats (Berkenbosch et al. 1990; Gaul et al. 1992; Gegelashvili et al. 1994), non‐human primates (Sil et al. 2020), and humans (Schmechel et al. 1988; Wolozin et al. 1992). High‐resolution single‐cell and single‐nucleus transcriptomic datasets confirm that *APP* transcription is a highly preserved feature of the astroglial lineage across phylogenies (Chen et al. 2024; Gabitto et al. 2024). These transcriptomic profiles match biochemical and immunohistochemical studies demonstrating abundant APP protein in astrocytes from mice (Trapp and Hauer 1994), rats (Berkenbosch et al. 1990; Card et al. 1988; Kawarabayashi et al. 1991), non‐human primates (Silhol et al. 1996), and humans (Stern et al. 1989; Tagawa et al. 1993; Tate‐Ostroff et al. 1989). Crucially, these analyses revealed a distinct cell‐type‐specificity: whereas neurons predominantly express the KPI‐deficient APP_695_ isoform, astrocytes mainly express the KPI‐containing APP_751_ and APP_770_ variants (Figure 1) (Gaul et al. 1992; Gegelashvili et al. 1994; LeBlanc et al. 1991; Rohan de Silva et al. 1997; Wolozin et al. 1992).

In the human brain, astrocytic APP is readily detected within the subpial and deep white matter during development, persisting broadly throughout the adult cerebrum and cerebellum (Nakamura et al. 1992; Takashima et al. 1990; Yamazaki et al. 1993). In AD tissue, this expression undergoes pronounced spatial reorganization, aligning tightly within the hypertrophic astroglial processes that form the glial barrier surrounding mature senile plaques (Martin et al. 1994; Yamaguchi et al. 1992). At the cellular level, APP distributes through astroglial cell bodies and processes, including perisynaptic leaflets and the perivascular endfeet wrapped around cerebral capillaries (Yamazaki et al. 1993). Subcellularly, APP transits through the constitutive secretory pathway, localizing across the endoplasmic reticulum, Golgi complex, plasma membrane, endosomal/lysosomal system, and specialized α1β1 integrin‐associated focal adhesion complexes (Kim et al. 2023; Yamazaki et al. 1997; Young et al. 1999). Astrocytes also express the APP homologous proteins amyloid precursor protein‐like protein 1 (APLP1) and 2 (APLP2), though their cell‐type‐specific trafficking profiles remain incompletely defined (Bayer et al. 1997; Crain et al. 1996).

### 2.2 APP Processing in Astrocytes

APP undergoes constitutive, sequential proteolytic cleavage by distinct secretase complexes to yield various bioactive fragments (Weidemann et al. 1989). In the non‐amyloidogenic pathway, α‐secretase—primarily a disintegrin and metalloproteinase domain‐containing protein 10 (ADAM10)—cleaves APP within the Aβ sequence, releasing a neuroprotective soluble fragment, soluble APPα (sAPPα), alongside a membrane‐bound C‐terminal fragment, C83 (Lammich et al. 1999). Subsequently, intramembrane cleavage of C83 by the γ‐secretase complex—comprising presenilin‐1 (PSEN1), nicastrin, anterior pharynx‐defective 1 (APH‐1), and presenilin enhancer 2 (PEN‐2)—releases the non‐toxic p3 fragment and the APP intracellular domain (AICD) (Francis et al. 2002; Haass et al. 1993; Takasugi et al. 2003). Conversely, the amyloidogenic pathway is initiated by β‐site APP cleavage enzymes 1 or 2 (BACE1, BACE2), which cleave APP at the N‐terminus of the Aβ domain, shedding soluble APPβ (sAPPβ) and retaining the C‐terminal fragment C99 (Fiore et al. 1995). The C99 fragment is then processed by γ‐secretase via sequential carboxypeptidase cleavages to yield Aβ and AICD. Notably, astrocytes rely significantly on BACE2 for this processing step; this enzyme is highly enriched in the non‐neuronal lineage and can serve as an amyloidogenic or protective secretase depending on cellular context (Bettegazzi et al. 2011).

Astrocytes express all essential machinery required for these processing cascades, including ADAM10 (Al‐Atrache et al. 2019; Grolla et al. 2013; O'Sullivan et al. 2016), BACE1 (Al‐Atrache et al. 2019; Grolla et al. 2013; Leuba et al. 2005; Rossner et al. 2005), and γ‐secretase components PSEN1 (Al‐Atrache et al. 2019; Grolla et al. 2013; Huynh et al. 1997; Nadler et al. 2008; Satoh and Kuroda 1999), nicastrin (Nadler et al. 2008), APH‐1 (Batiuk et al. 2020; Uhlen et al. 2015; Zhou et al. 2020), and PEN2 (Thul and Lindskog 2018). Genetic models utilizing selective neuronal deletions of γ‐secretase demonstrate that non‐neuronal cells produce sufficient Aβ load to drive parenchymal plaque deposition, suggesting that the astrocytic secretase machinery contributes directly to the total cerebral amyloid pool (Veeraraghavalu et al. 2014).

Astrocytes produce and secrete APP proteolytic fragments constitutively, although baseline Aβ yields vary across different experimental systems (Busciglio et al. 1993; Fukumoto et al. 1999; LeBlanc et al. 1997), reflecting a complex homeostatic balance across neural cell types (Oberstein et al. 2015). Under stress, however, reactive astrocytes exhibit coordinated upregulation of processing enzymes (Al‐Atrache et al. 2019; Hong et al. 2003; Liu et al. 2013; Nadler et al. 2008; Rossner et al. 2005; Wang et al. 2011; Zhao et al. 2011) in tandem with elevated baseline APP expression. This reactive transition shifts the equilibrium toward amyloidogenic processing, causing increased sAPPβ generation (Hong et al. 2003) and enhanced Aβ secretion (Blasko et al. 2000; Komatsu et al. 2022; Zhao et al. 2011) that directly fuels plaque accumulation (Liu et al. 2017; Nagele et al. 2003).

### 2.3 Functional Diversity of APP‐Derived Fragments

Importantly, APP‐derived metabolites possess distinct, and occasionally opposing, biological activities. Full‐length APP participates in cell adhesion, intracellular trafficking, and stress‐responsive signaling (Muller et al. 2017). Soluble APPα generally exhibits neurotrophic and homeostatic properties, whereas soluble APPβ appears to possess weaker trophic activity and can contribute to reactive signaling under pathological conditions (Richter et al. 2018; Ring et al. 2007). The AICD is implicated in transcriptional regulation and inflammatory signaling, although its specific astrocytic functions remain incompletely characterized (Cao and Sudhof 2001; Kimberly et al. 2001). In contrast, extracellular Aβ potently modulates astrocyte reactivity, calcium signaling, inflammatory mediator release, and metabolic dysfunction (Abramov et al. 2003; Abramov et al. 2004; LaRocca et al. 2021). Consequently, APP signaling does not comprise a uniform pathway but rather represents a family of interconnected signaling mechanisms mediated by distinct proteolytic products.

### 2.4 KPI‐Containing APP Isoforms in Astrocytes

The dominance of KPI‐containing APP variants (Oltersdorf et al. 1989; Ponte et al. 1988) in astrocytes (LeBlanc et al. 1991) mirrors their enrichment in AD brain tissue (Johnson et al. 1988; Matsui et al. 2007). Through the extracellular KPI domain, these isoforms regulate local proteolytic cascades by modulating serine proteases (Van Nostrand et al. 1989) involved in coagulation (Smith et al. 2004), kallikrein signaling (Petersen et al. 1994), plasmin activation (Tucker et al. 2000), and immune cell regulation (Hook et al. 1999).

Compared to the neuron‐specific APP_695_ variant, KPI‐containing isoforms exhibit distinct, context‐dependent processing kinetics. While some studies report restricted amyloidogenic processing (Belyaev et al. 2010), others demonstrate elevated Aβ_42_ secretion (Ho et al. 1996). Mechanistically, the KPI domain promotes cell‐surface homodimerization, which can favor non‐amyloidogenic α‐secretase cleavage (Ben Khalifa et al. 2012). Conversely, disrupting this dimerization footprint reduces both sAPPβ and Aβ secretion, indicating that dimerization also stabilizes essential β‐secretase interactions (So et al. 2012). In the human AD brain, upregulation of KPI‐containing isoforms strongly correlates with glial fibrillary acidic protein (GFAP) expression, sAPPα levels, and total Aβ burden (Matsui et al. 2007). Taken together, these findings suggest that the metabolic fate of KPI‐bearing APP is heavily dictated by cell‐type specific membrane trafficking, surface retention times, and endosomal sorting dynamics (Haass et al. 1991; Young et al. 1999).

### 2.5 Astrocytic Uptake and Clearance of Aβ

Beyond their capacity for synthesis, astrocytes serve as major clearance sinks for extracellular Aβ, dynamically balancing production and elimination under the transcriptional control of BACE1 (Zhou et al. 2023). Astrocytes preferentially internalize oligomeric Aβ over highly fibrillar aggregates, which resist efficient degradation and persist intracellularly (Nielsen et al. 2010; Sollvander et al. 2016). Post‐mortem analyses demonstrate substantial astrocytic Aβ accumulation during physiological aging (Funato et al. 1998), clinical AD (Kurt et al. 1999; Thal et al. 1999; Yamaguchi et al. 1998), and hereditary cerebral hemorrhage with amyloidosis (Maat‐Schieman et al. 2004). Furthermore, in vivo infusion and cellular transplantation assays confirm that astrocytes, rather than microglia, act as the primary long‐term repositories for internalized Aβ (Matsunaga et al. 2003; Nielsen et al. 2009; Perez et al. 2010; Pihlaja et al. 2008).

Intracellular degradation of astrocytic Aβ is driven by neprilysin (NEP), insulin‐degrading enzyme (IDE), and matrix metalloproteinases 2 and 9 (Yamamoto et al. 2013; Yamamoto et al. 2018; Yin et al. 2006). Astrocytic NEP expression is regulated by insulin‐like growth factor (IGF) signaling (Yamamoto et al. 2018), forkhead box O (FOXO) transcription factors (Du et al. 2021), and transcription factor EB (TFEB) (Xiao et al. 2014). In chronic disease states, endocytic transport networks fail; specifically, dynein‐dependent trafficking deficits stall degradation, driving Aβ accumulation within enlarged, dysfunctional astrocytic endosomes (Kimura et al. 2007; Lee et al. 2015).

Apolipoprotein E (ApoE), the primary genetic risk factor for late‐onset sporadic AD (Strittmatter et al. 1993), critically regulates this clearance axis. Aβ extensively colocalizes with ApoE‐positive astrocytic compartments in the AD brain (Utter et al. 2008), and ApoE deficiency impairs Aβ clearance kinetics (Koistinaho et al. 2004), while exacerbating Aβ‐mediated inflammatory responses (Hu et al. 1998). Astrocytes expressing the pathogenic ApoE ε4 allele exhibit defective Aβ uptake, compromised lysosomal clearance, and disrupted autophagy (Simonovitch et al. 2016). This receptor‐mediated clearance is driven by low‐density lipoprotein receptor (LDLR) and lipoprotein receptor‐related protein 1 (LRP1), whose downregulation exacerbates—and overexpression rescues—parenchymal Aβ accumulation (Basak et al. 2012; Liu et al. 2017).

In summary, reactive astrocytes operate at metabolic crossroads, coordinating receptor‐mediated uptake with intracellular degradation pathways. Deficits in these astrocytic clearance mechanisms promote extracellular Aβ stagnation and accelerate amyloid pathology (Basak et al. 2012; Koistinaho et al. 2004; Liu et al. 2017; Simonovitch et al. 2016; Yamamoto et al. 2013); though the precise relative contributions of astrocytic, microglial, and perivascular clearance pathways remain an active area of investigation.

## 3 Stressors Increase APP and Its Fragments in Astrocytes

**FIGURE 2:**
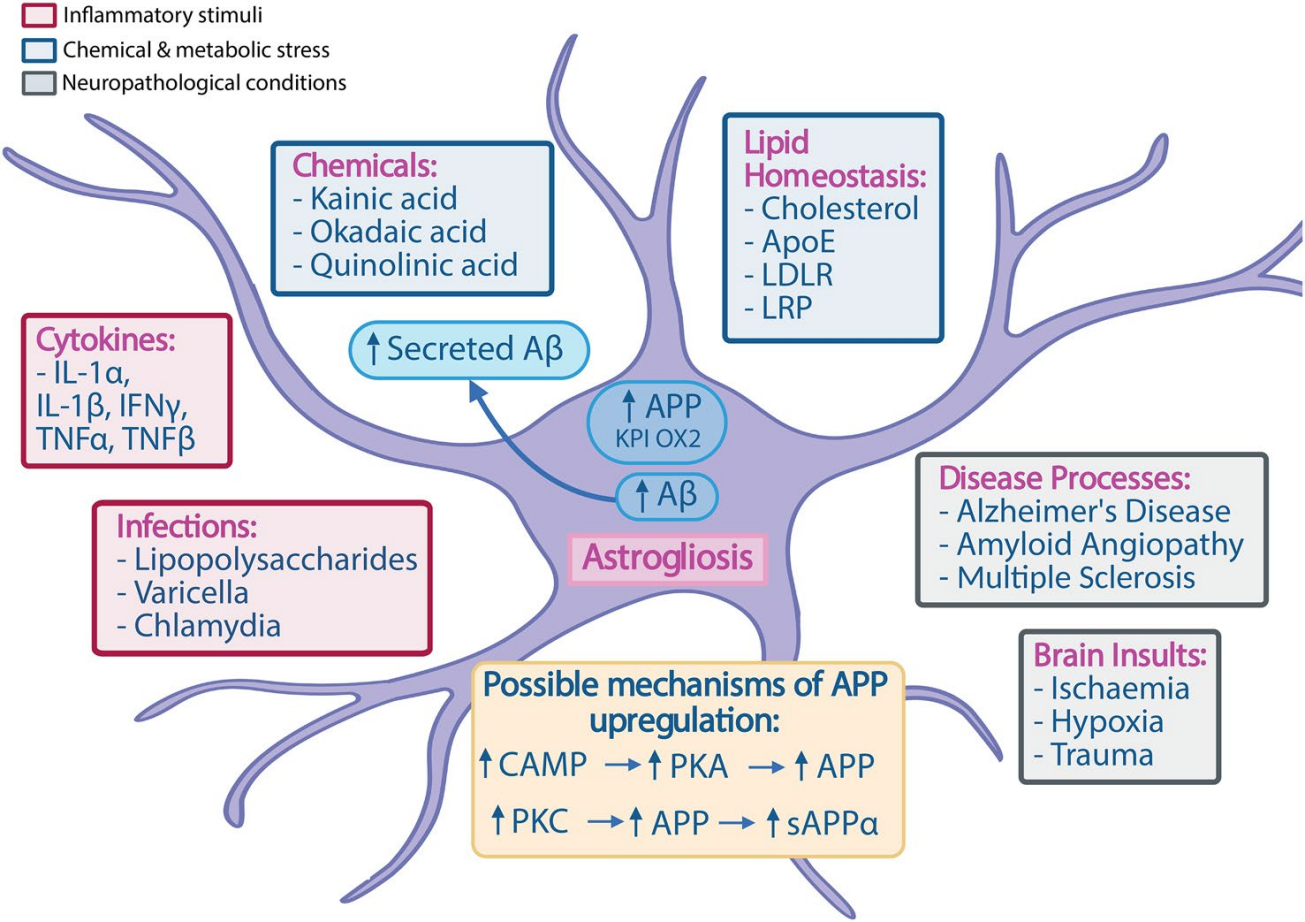
Stressor‐dependent regulation of astrocytic APP. Inflammatory, metabolic excitotoxic, and mechanical stressor promote astrocyte reactivity and increase APP expression and processing through cAMP/PKA‐ and PKC‐dependent signaling pathways. ApoE = apolipoprotein E; APP = amyloid precursor protein; Aβ = amyloid‐β peptides; cAMP = cyclic adenosine monophosphate; IFNγ = interferon γ; IL‐1α = interleukin 1α; IL‐1β = interleukin 1β; KPI = Kunitz‐type protease inhibitor domain, OX2 = MRC OX2 antigen; LDLR = low‐density lipoprotein receptor, LRP = lipoprotein receptor‐related protein 1; PKA = protein kinase A, PKC = protein kinase C; sAPPα = secreted α cleaved APP fragment; TNFα = tumor necrosis factor α, TNFβ = tumor necrosis factor β.

A broad spectrum of physiological and pathological insults converges to upregulate astrocytic APP transcription and amyloidogenic processing (Figure 2). Exposure to bacterial endotoxins such as lipopolysaccharide (LPS), excitotoxic agents (e.g., kainic acid, okadaic acid, quinolinic acid), traumatic brain injury (TBI), pro‐inflammatory cytokines, ischemic stroke, viral infections, and hypercholesterolemia all drive significant elevations in astrocytic APP expression (Table 1). This stereotypical response is accompanied by a coordinated shift toward amyloidogenic cleavage, accelerating the production of sAPPβ and Aβ. These data indicate that altered APP processing is a highly conserved component of the astroglial stress response (Blasko et al. 2000; Bubak et al. 2020; Burton et al. 2002; Komatsu et al. 2022; Ourdev et al. 2019; Zhao et al. 2011). Rather than merely serving as a passive byproduct, these metabolites can amplify APP‐dependent signaling within the local microenvironment while simultaneously compounding the local amyloid burden. Importantly, APP upregulation occurs early in the astrocyte stress cascade, frequently preceding classical morphological signs of reactivity (Banati et al. 1995; Hall et al. 1995; Palacios et al. 1995; Siman et al. 1990).

**TABLE 1:** Astrocytes translate brain stress into APP signaling.

Treatment	Model	Outcome	References
*LPS*			
Intraventricular LPS injections, brain stabbing or TBI, exposure to LPS, IL‐1 or viruses	Rats, mice, human cortical astrocytes, rat astrocytes, human astrocytoma	Reactive astrocytes, increase in APP, PS1, IL‐1β, IL‐6, IL‐12, TNFα, TGFβ1, INFγ, G‐CSF, iNOS, HSP70, mn‐SOD	Altstiel and Sperber (1991); Forloni et al. (1997); Hauss‐Wegrzyniak et al. (1998); Nadler et al. (2008); Sugaya et al. (1998); Velezmoro Jauregui et al. (2024)
*Cytokines*			
Intracerebral IL‐1, IL‐1β or IFNγ injections, exposure, TGFβ, IL‐1α, IL‐1β, TNFα or IFNγ	Rats, mice, human astrocytes, astrocytes, human astrocytoma, U373 MG astrocytoma, mouse astrocytes	Reactive astrocytes, increase in APP, in KPI containing APP, sAPPα, C‐terminal fragments, secreted Aβ, ADAM‐10, ADAM‐17, BACE‐1, antichymotrypsin	Amara et al. (1999); Bandyopadhyay et al. (2006); Burton et al. (2002); Cho et al. (2007); Gitter et al. (2000); Gray and Patel (1993); Rogers et al. (1999); Sheng et al. (1996); Zhao et al. (2011)
*Excitotoxicity*			
Intraventricular and systemic kainic acid injections, basal nucleus okadaic acid injections, striatal quinolinic acid injections, systemic chloroquine injections, exposure to kainic acid	Rats, mice, astrocytes, rat astrocytes, U‐373 astrocytoma	Reactive astrocytes, increase in APP, α and β C‐terminal fragments, Aβ, intracellular Aβ, KPI containing APP, calpain I	Arendt et al. (1994); Kodam et al. (2019); Mielke et al. (1997); Ourdev et al. (2019); Shoham and Ebstein (1997); Siman et al. (1990); Sola et al. (1993); Topper et al. (1995)
*Ischemia*			
4 vessel, carotid or middle cerebral artery occlusions, cardiac arrest, hypoxia	Rats, gerbils, cortical astrocytes	Reactive astrocytes, increase in APP, PS1, Aβ	Banati et al. (1995); Hall et al. (1995); Kalaria et al. (1993); Pluta (2002); Smith et al. (2004)
*TBI*			
Needle stab wound, TBI	Rats	Reactive astrocytes, increase in APP, BACE‐1	Blasko et al. (2004); Otsuka et al. (1991)
*Infections*			
Chlamydia, varicella zoster virus	Spinal astrocytes, astrocytoma	Reactive astrocytes, increase in APP, BACE1, PS1, Aβ	Al‐Atrache et al. (2019); Bubak et al. (2020)
*Cholesterol*			
Cholesterol	Astrocytes	Reactive astrocytes, increase in APP, BACE‐1 interaction, reduced sAPPα	Avila‐Munoz and Arias (2015); Galbete et al. (2000); Xiu et al. (2006)
*Other*			
Cuprizone, hyperammonemia, ATP, norepinephrine, isoproterenol	Mice, Tg2576 mice, astrocytes, rat astrocytes	Reactive astrocytes, increase in APP, APP in astrocytes far from senile plaques	Clarner et al. (2011); Heiland et al. (2019); Komatsu et al. (2022); Lee et al. (1997); Tran (2011)

Abbreviations: ADAM‐10 = A disintegrin and metalloproteinase domain‐containing protein 10, ADAM‐17 = A disintegrin and metalloproteinase domain‐containing protein 17, Aβ = amyloid‐β peptides, BACE‐1 = beta‐site APP cleaving enzyme 1, G‐CSF = granulocyte colony stimulating factor, HSP70 = 70 kiloDalton heat shock proteins, IL‐12 = interleukin 12, IL‐1α = interleukin 1α, IL‐1β = interleukin 1β, IL‐6 = interleukin 6, INFγ = interferon γ, iNOS = inducible nitric oxide synthase, KPI = Kunitz type protease inhibitor domain, LPS = LIPOPOLYSACCHARIDE, mn‐SOD = manganese superoxide dismutase, PS1 = presenilin‐1, sAPPα = α cleaved secreted extracellular domain of APP, TBI = traumatic brain injury, TGFβ1 = tumor growth factor β1, TNFα = tumor necrosis factor α.

At the molecular level, cytokine‐driven JAK/STAT3 signaling coordinates this reactive astrocyte transformation through a bidirectional loop linking APP metabolism to astrocyte activation states (Velezmoro Jauregui et al. 2024). Specifically, proinflammatory cytokines activate STAT3 (O'Callaghan et al. 2014; Trindade et al. 2020; Zamanian et al. 2012), while elevated APP expression reciprocally enhances cytokine release and sustained STAT3 phosphorylation (Ben Haim et al. 2015; Ceyzeriat et al. 2018). Downstream, activated STAT3 directly promotes amyloidogenic β‐secretase processing (Wen et al. 2008), establishing a chronic pathogenic cycle.

This stress response manifests across distinct spatial and morphological dimensions in the degenerating brain (Beauquis et al. 2013). Astrocytes proximal to injury sites or amyloid plaques adopt a hypertrophic, reactive phenotype, whereas more distant populations frequently undergo progressive atrophy (Habib et al. 2020; Hasel et al. 2021; Mallach et al. 2024; Olabarria et al. 2010; Wanner et al. 2013). Disrupting this spatial heterogeneity or artificially dampening the reactive phenotype paradoxically exacerbates both amyloid and axonal pathology (Ceyzeriat et al. 2018; Kraft et al. 2013). Mechanistically, reactive astrocytes concurrently upregulate APP and its associated proteolytic machinery, directly accelerating Aβ production (Al‐Atrache et al. 2019; Banati et al. 1995; Gehrmann et al. 1995; Hartlage‐Rubsamen et al. 2003; Liu et al. 2017; Martin et al. 1994; Rossner et al. 2005; Yamaguchi et al. 1992). Conversely, atrophic astrocytes exhibit compromised endocytosis and disrupted APP trafficking networks (Kulijewicz‐Nawrot et al. 2012; Liu et al. 2017; Verkhratsky et al. 2019). While hypertrophy predominates during acute insults and prodromal disease phases, transcriptional dysregulation of *APP* tends to persist throughout chronic neurodegeneration (Banati et al. 1995; Bellaver et al. 2023; Das et al. 2020; Gehrmann et al. 1995; Rodriguez‐Vieitez et al. 2016). How intra‐regional astrocyte heterogeneity further dictates localized APP processing and Aβ handling dynamics remains an active focus of exploration (Bayraktar et al. 2020; Bradley et al. 2019; Hochstim et al. 2008; Tsai et al. 2012).

## 4 Mechanisms Regulating APP Upregulation in Astrocytes

The molecular pathways governing astrocytic APP upregulation are tightly coupled to second‐messenger systems. Intracellular elevation of cyclic adenosine monophosphate (cAMP) activates protein kinase A (PKA), which directly drives APP transcription while inducing reactive morphological transformations (Gegelashvili et al. 1996; Lee and Wurtman 1997). These transcriptional effects are highly sensitive to transmembrane Na^+^/K^+^ ionic gradients, directly linking homeostatic ionic shifts to APP expression (Abe and Saito 2001). Consistently, pharmacological activation of PKA enhances, whereas its targeted inhibition suppresses, astrocytic APP expression (Lee et al. 1999).

Inflammatory mediators such as prostaglandin E_2_ (PGE_2_) exploit this same cascade; by elevating intracellular cAMP, PGE_2_ concurrently increases both *APP* and *GFAP* expression—an effect completely blunted by cyclosporine administration (Lee et al. 1997). Parallel to the PKA axis, activation of protein kinase C (PKC) by phorbol esters modulates post‐translational sorting, shifting the equilibrium toward non‐amyloidogenic processing and accelerating APP sAPPα secretion (Lee and Wurtman 1997). Together, the PKA and PKC arms integrate extracellular stress signals with APP metabolism. By co‐regulating APP expression and enzymatic cleavage, these signaling cascades couple the state of astrocytic reactivity directly to downstream amyloid dynamics and APP‐dependent intercellular communication.

## 5 Effects of APP and Its Fragments on Astrocytes

### 5.1 Effects of Full‐Length APP

**FIGURE 3:**
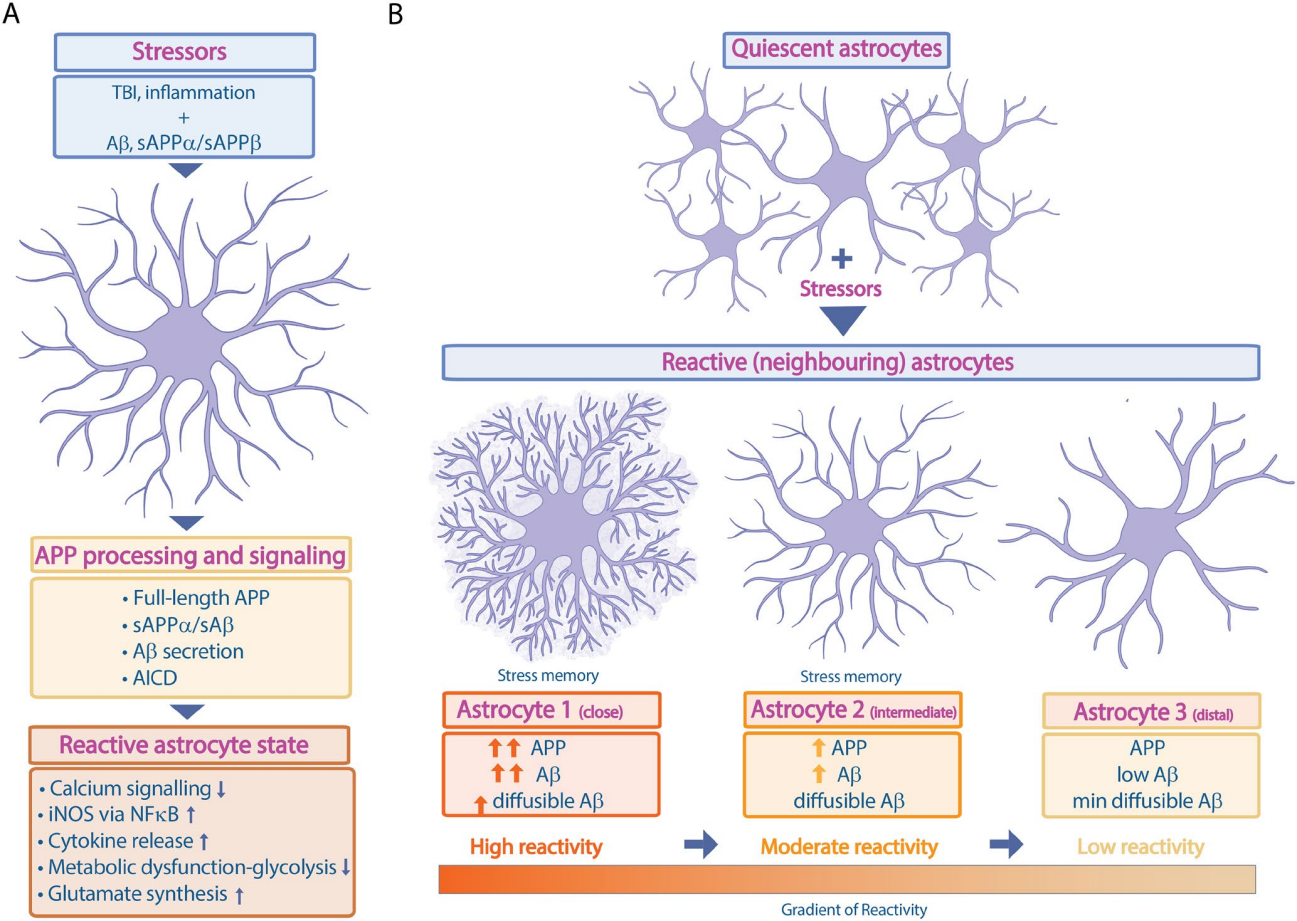
APP/Aβ‐driven astrocyte reactivity. APP, APP fragments, and Aβ promote astrocyte activation, cytokine release, metabolic dysfunction, and impaired synaptic support, sustaining chronic neuroinflammation. (A) Cellular effect of APP/Aβ. (B) APP‐dependent gradients of reactive astrocytes. APP = amyloid precursor protein, Aβ = amyloid‐β peptides, iNOS = inducible nitric oxide synthase, secreted APPα = secreted α cleaved APP fragment, secreted APPβ = secreted β cleaved APP fragment, TBI = traumatic brain injury, NFκB = nuclear factor kappa‐light‐chain‐enhancer of activated B cells.

Full‐length APP (holo‐APP) directly regulates the induction and maintenance of the astroglial reactive state. Exposing primary astrocytes to full‐length holo‐APP induces a reactive transcriptional profile that closely recapitulates the molecular signature elicited by acute LPS exposure (Figure 3a) (Velezmoro Jauregui et al. 2024). Astrocytic overexpression of APP is sufficient to trigger autonomous cytokine production and reactive state acquisition in vivo. Conversely, conditional ablation of astrocytic APP dampens these inflammatory responses (Velezmoro Jauregui et al. 2024). Consistently, reactive astrocytes in transgenic APP mouse models show pronounced immunoreactivity for IL‐1β, TGFβ, and interleukin‐10 (IL‐10) (Apelt and Schliebs 2001; Benzing et al. 1999; Tehranian et al. 2001), reinforcing a role of full‐length APP in orchestrating astroglial inflammatory signaling.

Loss of perivascular aquaporin‐4 (AQP4) polarization is another hallmark of AD, characterized by redistribution of AQP4 from astrocytic endfeet to non‐endfoot membrane domains (Yang et al. 2011; Zeppenfeld et al. 2017). Beyond its canonical link to Aβ pathology, APP handles crucial cell‐autonomous roles in regulating astrocyte activation, adhesion signaling, and intracellular trafficking (Kim et al. 2023). While direct biochemical regulation of AQP4 by APP remains to be demonstrated, APP‐dependent alterations in astrocyte morphology and structural remodeling are positioned to modulate perivascular AQP4 anchoring, thereby indirectly impacting glymphatic clearance efficiency (Kitchen et al. 2020; Salman et al. 2022).

### 5.2 Effects of Aβ Species

Astrocytes are continuously exposed to elevated concentrations of Aβ species due to their intimate structural association with senile plaques. Focal parenchyma injection of Aβ_42_ oligomers induces rapid, widespread reactive astrogliosis in vivo, whereas systemic Aβ_42_ immunization attenuates astrocyte reactivity in transgenic APP models, confirming that extracellular Aβ dynamics directly drive astroglial reactive states (Perez et al. 2010; Schenk et al. 1999).

Human positron emission tomography (PET) studies indicate that astrogliosis is an early feature of AD pathogenesis. This reactive phenotype precedes symptom onset in familial AD and scales with amyloid burden in individuals with mild cognitive impairment (Calsolaro et al. 2021; Carter et al. 2012; Rodriguez‐Vieitez et al. 2016). Consistent with these clinical observations, reactive astrogliosis precedes overt amyloid deposition in several APP mouse models (Rodriguez‐Vieitez et al. 2015), though the exact temporal alignment between astrogliosis, amyloid accumulation, and cognitive decline remains model‐dependent (Ameen‐Ali et al. 2019).

In vitro, Aβ exposure directly triggers astrocyte reactivity, accelerating cytokine and chemokine release, disrupting metabolic fluxes, and reducing glutamate uptake capacity. This state is further characterized by altered Ca^2+^ homeostasis and the induction of inducible nitric oxide synthase (iNOS), which drives nitric oxide (NO) production (Figure 3a, Table 2). Aβ reciprocally upregulates astrocytic APP expression, establishing a positive feedback loop that amplifies local APP/Aβ‐dependent signaling cascades (Carlson et al. 2000; Moreno‐Flores et al. 1998). Collectively, these autonomous astroglial defects converge to promote neuroinflammation, metabolic exhaustion, excitotoxicity, aberrant gliotransmission, and nitrosative stress within the neural microenvironment.

**TABLE 2:** Aβ‐induced astrocyte state transitions.

Types of Aβ	Model	Outcome	References
*Morphology*			
Aβ, Aβ40, Aβ42, aggregated Aβ, Aβ42 oligomers, Aβ25‐35 and Aβ fragments	Cultured astrocytes, cortical astrocytes, rat astrocytes, rat cortical and hippocampal astrocytes	Reactive astrocytes	Akama and Van Eldik (2000); Chow et al. (2010); Hou et al. (2011); Hu et al. (1998); Jalonen et al. (1997); Kato et al. (1997); Matos et al. (2012); Meske et al. (1998); Moreno‐Flores et al. (1998); Pike et al. (1994); Pike et al. (1996); Salinero, Moreno‐Flores, Ceballos, and Wandosell (1997); Salinero, Moreno‐Flores, and Wandosell (1997); Schubert et al. (2009)
*Cytokines and chemokines*			
Aβ, Aβ42, aggregated Aβ40 and Aβ42, Aβ and Aβ42 oligomers	Cultured astrocytes, adult astrocytes, cortical astrocytes, rat astrocytes, rat hippocampal astrocytes, U‐373 MG astrocytoma	Increase in proinflammatory cytokines, increase in IL‐1β, IL‐8, TNFα, MCP‐1, some changes prevented by 17β estradiol	Akama and Van Eldik (2000); Araujo and Cotman (1992); Carlson et al. (2000); Hou et al. (2011); Hu et al. (1998); Johnstone et al. (1999); Schulte‐Herbruggen et al. (2007); Smits et al. (2002); Valles et al. (2010); White et al. (2005)
*Metabolism*			
Aβ, Aβ42, Aβ42 oligomers, Aβ42 fibrils	Cultured astrocytes, neonatal rat astrocytes, rat astrocytes, hiPSC‐derived astrocytes w/wo FAD mutations	Reduced cellular redox and metabolic activity, increase in ROS, decrease in HIF‐1α and glucose, pyruvate and lactate use, increase in glycogen, change in glycolysis, mitochondrial fusion, then swelling and fission	Abe et al. (1997); Johnstone et al. (1999); Kerokoski et al. (2001); Salcedo et al. (2024); Schubert et al. (2009); Tarczyluk et al. (2015); Zysk et al. (2023)
*Glutamate*			
Aβ, Aβ42, aggregated Aβ, Aβ25‐35	Cultured astrocytes, muse and rat astrocytes, rat cortical and hippocampal astrocytes, hippocampal slices, acute brain slices from Tg AD mouse model, 3× Tg AD mouse mice	Decrease in glutamate uptake, increase in α7 receptor‐mediated glutamate release, change in glutamine synthase, decrease in EAAT1 and EAAT2 levels, changes in functional glutamate transporters and AMPA/kainite receptors, Aβ effects enhanced by A(2A) receptor agonists, which increase Cx43 and ATP release	Abe and Saito (2000); Fernandez‐Tome et al. (2004); Harris et al. (1996); Madeira et al. (2021); Matos et al. (2012); Matos et al. (2008); Olabarria et al. (2011); Parpura‐Gill et al. (1997); Peters et al. (2009); Pike et al. (1996); Scimemi et al. (2013); Talantova et al. (2013)
*Calcium*			
Aβ, Aβ40, Aβ42, Aβ25‐35, Aβ fragments	Cultured astrocytes, cortical astrocytes, rat cortical and hippocampal astrocytes, hiPSC‐derived astrocytes w PS1δE9 mutation, mixed neuron astrocyte cultures	Altered Ca homeostasis, intracellular Ca transients, increased amplitude, velocity and frequency of Ca transients, increase in Ca influx	Abramov et al. (2003); Chow et al. (2010); Daschil et al. (2014); Haughey and Mattson (2003); Jalonen et al. (1997); Lee et al. (2014); Lim et al. (2013); Meske et al. (1998); Oksanen et al. (2017)
*Nitric oxide*			
Aβ, Aβ42, Aβ25‐35, oligomeric and fibrillar Aβ	Cultured astrocytes, rat and human astrocytes, midbrain astrocytes, intracerebral injection	Increase in iNOS and NO production via NFκB, IL‐1β or TNFα, potentiated by IFNg, NFκB and PKC induce COX2 and PGE2, decrease in cGMP in response to NO due to decrease in sGC	Akama et al. (1998); Akama and Van Eldik (2000); Baltrons et al. (2002); Blanco et al. (2010); Casal et al. (2004); Dodel et al. (1999); Hu et al. (1998); Hull et al. (2006); Rossi and Bianchini (1996); Valles et al. (2010); White et al. (2005)
*Other*			
Aβ, Aβ42, Aβ25‐35, Aβ oligomers, Tg2576 mice	Cultured astrocytes, mouse and rat astrocytes, mixed neuron astrocytes cultures, Aβ infused rat cortex, APP23/GFAP‐TK mice, 5× FAD mouse mode, Down syndrome tissue	Upregulated K(V)3.4, altered AChE glycosylation, attenuated synapse formation and transmission, decrements in EPSC, clathrin and dynamin independent endocytic vesicles transition faster from early to late endosomes, interrupted Cx43 endocytosis, activated JNK and p38K mediate astrocyte proliferation, increase in CB(2), YKL40 and DBI, ganciclovir ablates proliferating astrocytes, increase in Golgi apparatus cholesterol and caveolin	Boscia et al. (2017); Fodero et al. (2002); Jiang and Chen (2009); Katsouri et al. (2020); Kawano et al. (2017); Maulik et al. (2020); Nunez et al. (2008); Saha et al. (2020); Tokay et al. (2005); Zeng et al. (2023)

Abbreviations: Aβ = amyloid‐β peptides, A(2A) receptor = adenosine A2A receptor, AChE = acetylcholine esterase, CB(2) = cannabinoid receptor CB(2), cGMP = cyclic guanosine monophosphate, COX2 = cyclooxygenase 2, Cx43 = Connexin 43, DBI = diazepam binding inhibitor, EEAT1 = excitatory amino acid transporter 1, EEAT2 = excitatory amino acid transporter 2, EPSC = excitatory post‐synaptic current, HIF‐1α = hypoxia‐inducible factor 1α, IL‐1β = interleukin 1β, IL‐8 = interleukin 8, INFγ = interferon γ, iNOS = inducible nitric oxide synthase, JNK = c‐Jun N‐terminal kinases, MCP‐1 = monocyte chemoattractant protein 1, NFκB = nuclear factor kappa‐light‐chain‐enhancer of activated B cells, NO = nitric oxide, PGE2 = prostaglandin E2, PKC = protein kinase C, p38 = p38 mitogen activated protein kinases, ROS = reactive oxygen species, sGC = soluble guanylyl cyclase, TNFα = tumor necrosis factor α, YKL40 = chitinase‐3‐like protein 1.

### 5.3 Effects of Other APP Fragments

In neural progenitors derived from the rat hippocampal subgranular zone, both sAPPα and sAPPβ increase GFAP immunoreactivity, promote cell proliferation, and enhance survival, suggesting that these secreted fragments actively modulate astrocyte differentiation and astrogliosis (Baratchi et al. 2012). Consistent with this, exposure to sAPPα directly alters the astrocyte proteome and secretome, triggering distinctive changes in cell morphology and vesicle trafficking dynamics (Peppercorn et al. 2023).

Although the cell‐autonomous functions of the AICD remain less defined, particularly within the macroglial lineage, emerging evidence indicates that AICD is sufficient to induce an astroglial reactive state. This induction is driven through the activation of mitogen‐activated protein (MAP) kinase pathways and the nuclear factor kappa‐light‐chain‐enhancer of activated B cells (NF‐κB) signaling network (Bach et al. 2001).

## 6 An Integrative Model of Astrocytic APP Signaling

Several factors validate the physiological plausibility of an astrocytic APP signaling network. Astrocytes constitutively express both holo‐APP and its canonical processing machinery. In response to diverse inflammatory, metabolic, excitotoxic, and mechanical stressors, these macroglia upregulate APP expression and shift toward amyloidogenic cleavage. The resulting metabolites, particularly Aβ, directly alter astroglial physiology to reinforce reactive phenotypes. Crucially, this astrocytic transformation frequently precedes substantial parenchymal amyloid deposition in both transgenic models and clinical neuroimaging studies (Carter et al. 2012; Rodriguez‐Vieitez et al. 2016). Collectively, these findings indicate that astrocytic APP dynamics actively drive, rather than passively reflect, early disease pathogenesis.

We propose that astrocytic APP is not merely an inert catabolic precursor, but a stress‐responsive signaling molecule and metabolic integrator (Jin et al. 2024; Wu et al. 2023; Xu et al. 2017; Xu et al. 2018; Zhang et al. 2020). Within this framework, full‐length holo‐APP acts as an unmapped sensor for microenvironmental distress, whereas Aβ serves as a primary diffusible effector capable of propagating reactive states to neighboring astrocytes. Supplementary fragments—including sAPPα, sAPPβ, and AICD—exert additional homeostatic modulations that remain to be fully characterized (Figure 3b).

Chronic exposure to environmental and aging‐related insults progressively shifts astrocytes from homeostatic to neurodestructive states. This phenotypic transition accelerates amyloidogenic processing while simultaneously crippling receptor‐mediated Aβ clearance, driving localized amyloid accumulation (Blasko et al. 2000; Ries and Sastre 2016). This feed‐forward loop is integrated by multiple overlapping intracellular cascades, including STAT3 (Ben Haim et al. 2015; Ceyzeriat et al. 2018), NF‐κB (Bach et al. 2001; Johnstone et al. 1999), Toll‐like receptor (TLR) (Stewart et al. 2010; Walter et al. 2007), NLRP3 inflammasome activation (Couturier et al. 2016; Stewart et al. 2010), purinergic signaling (Delekate et al. 2014), and cell‐adhesion circuits (Kim et al. 2023; Wu et al. 1997). This autonomous network is further shaped by reciprocal intercellular crosstalk with microglia, neurons, and the neurovascular unit (Henstridge et al. 2019; Iadecola 2004; Nanclares et al. 2021; Patani et al. 2023). Consequently, sporadic AD pathogenesis may develop when stress‐induced astrocytic APP signaling breaks through local homeostatic control mechanisms, establishing self‐sustaining reactive astrocyte networks that accelerate neuroinflammation and neurodegeneration.

### 6.1 Relationship to Conventional AD Hypotheses

This astrocytic APP framework complements, rather than opposes, established neuron‐ and microglia‐centric models of AD (Hardy and Higgins 1992; Hong et al. 2016). While neurons remain a major source of synaptic Aβ and microglia drive chronic neuroinflammatory pruning, the present model positions astrocytic APP signaling as an upstream amplifier and spatial organizer of these distinct pathological arms.

Importantly, this model does not mandate that astrocytes serve as the primary source of total cerebral Aβ. Instead, even a modest shift in astroglial APP processing can exert disproportionate local effects on disease progression by permanently altering homeostatic, metabolic, and blood–brain barrier‐supportive astrocyte functions. Stress‐induced astrocytic APP activation therefore acts in parallel with neuronal, microglial, vascular, and tau‐dependent pathways, serving as a critical bridge linking early tissue stress to widespread neurodestructive cascades.

## 7 Outstanding Questions and Future Directions

Several major knowledge gaps must be resolved to validate and refine this proposed model of astrocytic APP signaling. First, the precise upstream molecular pathways mapping environmental and cellular stressors to astrocytic APP transcription, intracellular trafficking, and enzymatic cleavage remain incomplete. Specifically, the signaling cascades engaged by the astrocyte‐enriched KPI‐ and OX2‐containing APP variants require deeper mechanistic characterization. Second, it remains crucial to determine whether chronic cell‐autonomous reactivity directly cripples astrocytic degradation networks, effectively converting these cells from net clearance sinks into self‐sustaining sources of pathogenic Aβ. Third, identifying whether these APP‐dependent reactive states can be selectively reversed without disrupting vital homeostatic support of local circuits will be key to establishing the translational feasibility of this target axis.

Answering these questions will mandate cell‐type‐specific experimental manipulations paired with spatially resolved single‐cell multi‐omic profiling. Utilizing human iPSC‐derived astrocytes, three‐dimensional brain organoids, and astrocyte‐specific transgenic lines—alongside spatial proteomic and transcriptomic datasets—will be critical to bypass the limitations of traditional, neuron‐centric experimental models.

Finally, future studies must evaluate whether stress‐induced astrocytic APP signaling represents an AD‐specific vulnerability or a generalized macroglial stress program. This pathogenic signature may be shared across other disorders featuring prominent astroglial pathology, including ischemic stroke, glial tumors, demyelinating disease, or conditions with primary astrocytic drives such as autoimmune GFAP astrocytopathy (Fang et al. 2016; Kunchok et al. 2019; Toda et al. 2007), Alexander disease (Brenner et al. 2001), aging‐related tau astrogliopathy (ARTAG), and limbic predominant age‐related TDP‐43 encephalopathy (LATE) (Kovacs et al. 2016; Kovacs et al. 2017; Nelson et al. 2019).

## Author Contributions

**Sophie Coomes:** conceptualization, data curation, investigation, writing – original draft, visualization. **Gretsen Velezmoro Jauregui:** conceptualization, data curation, writing – original draft, writing – review and editing, investigation. **Alex Thatcher:** conceptualization, data curation, investigation. **Elizabeth Emmett:** conceptualization, data curation, investigation. **Tarun Kuruvilla:** investigation. **Vladimir Parpura:** writing – review and editing, supervision. **Jana Zelinkova:** conceptualization, data curation. **Maria Čarna:** conceptualization, visualization. **Robert Zorec:** writing – review and editing, supervision. **Jan Sebastian Novotny:** investigation. **Natalie Polakova:** investigation. **Kenneth L. Moya:** writing – review and editing. **Clara Limback‐Stokin:** writing – original draft, writing – review and editing, supervision. **Gorazd Bernard Stokin:** conceptualization, writing – review and editing, writing – original draft, supervision. **Nicole Oyelakin:** conceptualization, data curation, investigation. **David Morgan:** writing – review and editing. **Alexei Verkhratsky:** writing – review and editing, supervision.

## Funding

This study was funded by the European Union project Next Generation EU—Project National Institute for Neurological Research LX22NPO5107 by the Ministry of Education, Youth and Sports of the Czech Republic (GBS). V.P. work is supported by the National Natural Science Foundation of China and the Zhejiang Province People's Government Awards. R.Z. is supported by the Slovenian Research and Innovation Agency grants: P3‐310, J3‐50104, J7‐3153, J3‐2523, J4‐60077, I0‐0034 Celica, I0‐0048 Cipkebip, I0‐0022 UL, EU Interreg Italia‐Slovenia Immunocluster‐2, and Coherence.

## Conflicts of Interest

The authors declare no conflicts of interest.

## Data Availability

Data sharing not applicable to this article as no datasets were generated or analysed during the current study.
